# Fecal microbiota transplantation reduces inflammation and modulates gene expression in HIV-infected double humanized-BLT (dHu-BLT) mice on antiretroviral therapy

**DOI:** 10.3389/fimmu.2026.1773716

**Published:** 2026-06-03

**Authors:** Saroj Chandra Lohani, Chi Zhang, Subhra Mandal, Miaoyun Zhao, Yilun Cheng, Amanda E. Ramer-Tait, Qingsheng Li

**Affiliations:** 1 HIV Cure and Viral Diseases Center, The Wistar Institute, Philadelphia, PA, United States; 2 Nebraska Center for Virology, University of Nebraska-Lincoln, Lincoln, NE, United States; 3 School of Biological Sciences, University of Nebraska-Lincoln, Lincoln, NE, United States; 4 Department of Food Science and Technology, University of Nebraska-Lincoln, Lincoln, NE, United States; 5 Nebraska Food for Health Center, University of Nebraska-Lincoln, Lincoln, NE, United States

**Keywords:** antiretroviral therapy (ART), fecal microbiota transplantation (FMT), gut Inflammation, gut microbial dysbiosis, gut transcriptome, HIV, people living with HIV (PLWH)

## Abstract

**Introduction:**

Persistent immune activation and inflammation remain significant barriers to managing comorbidities in people living with HIV (PLWH) on suppressive antiretroviral therapy (ART). While ART substantially reduces plasma viral loads to an undetectable level, it fails to fully restore gut microbial homeostasis and prevent microbial translocation, a critical pathogenic contributor to systemic persistent immune activation and inflammation. To evaluate the potential of human fecal microbiota transplantation (FMT) as an adjunctive therapy to restore gut health and attenuate inflammation and immune activation in PLWH on ART, we utilized a double humanized-BLT (dHu-BLT) mouse model, featuring a functional human immune system and a human-like microbiome.

**Methods:**

Two groups of HIV-infected dHu-BLT mice were used in the study. One group received FMT in addition to ART, while the control group received ART alone. Using both a multi-omics approach (16S rRNA sequencing and RNA-seq) and an immune-based assay, we compared alterations in gut microbial composition, profiled transcriptomic changes in the intestinal tissue, and quantified markers of systemic immune activation and inflammation between the groups.

**Result:**

FMT supplementation in ART-treated mice increased the relative abundance of beneficial bacteria and modulated the transcriptomic profile of both human- and murine-related genes. Notably, genes associated with cellular structure and tissue maintenance, including *Mcpt4*, were upregulated, along with the extracellular matrix organization pathway predicted as the most strongly activated pathway in the FMT-supplemented group compared to ART alone. In contrast, genes and signaling pathways associated with inflammation were downregulated. Importantly, the FMT-supplemented group exhibited a significant reduction of plasma inflammatory markers, including CD62E, sCD14, sCD163, and FABP2, relative to the ART alone group.

**Conclusion:**

These results suggest that FMT may serve as a promising adjunctive strategy for mitigating systemic inflammation by improving gut health, thereby contributing to the reduction of comorbidities in PLWH on ART.

## 1 Introduction

HIV-1 (HIV) is treatable but remains incurable. Although antiretroviral therapy (ART) can effectively reduce the plasma viral load to an undetectable level, the damage to the gastrointestinal tract and microbiome is never fully restored to the state of an uninfected individual (1–3). Consequently, this gastrointestinal and microbial dysfunction is considered a major driver of systemic and persistent immune activation and chronic inflammation in people living with HIV (PLWH) on ART (4–7), thereby promoting non-AIDS-related comorbidities, including premature aging, cardiovascular disease, renal disease, liver disease, bone disease, and neurological conditions in aviremic PLWH on successful ART treatment (8–10). Hence, mitigating abnormal immune activation and inflammation represents an important interventional strategy for managing HIV-associated comorbidities and improving the quality of life in PLWH on durable ART-mediated viral suppression.

Numerous studies have shown that HIV infection induces a compositional and functional shift in the gut microbiota, characterized by the enrichment of bacterial populations capable of promoting inflammation and a depletion of healthy microbial communities (11–13). It is widely accepted that persistent immune activation and inflammation in HIV-infected individuals result from damage to the intestinal epithelial barrier and systemic translocation of microbial products, even among PLWH undergoing suppressive ART (4–6). Fecal microbiota transplantation (FMT) has emerged as a promising therapy for correcting gut dysbiosis, with encouraging efficacy in improving outcomes for gastrointestinal (GI) disorders such as recurrent *Clostridioides difficile* infection, irritable bowel syndrome, and ulcerative colitis (14, 15). Given that FMT primarily focuses on replenishing the gut microbial community, it is hypothesized to bring about clinically beneficial effects within the host’s GI tract through the control of “potentially harmful” microbes and the introduction of “beneficial” ones (14). This microbial reconstitution following FMT not only re-establishes microbial diversity but also modulates host transcriptional programs. FMT can induce epigenetic modifications, including changes in DNA methylation and histone modification, thereby promoting both intestinal and systemic homeostasis (16). Evidence from preclinical animal models and human clinical studies indicates that FMT alters the expression of genes associated with immune responses, inflammatory signaling, metabolic regulation, and epithelial barrier functions (17–20). In addition, a positive impact of FMT on restoring gut mucosal integrity and alleviating intestinal inflammation has been demonstrated in several studies (18–23). Together, these highlight the role of FMT in re-establishing host–microbiome homeostasis at both microbial and molecular levels.

The number of clinical studies of FMT in PLWH supports its safety and its ability to induce shifts in gut microbial composition; however, its effects on systemic immune activation and inflammation, and on transcriptomic changes in gut tissue, remain limited (23, 24). Clinical studies are often complicated by several variables, such as individual dietary habits, sexual behavior, type and duration of ART regimen, all of which may independently or in combination influence the gut microbiome and can mask the effects of FMT (24). Here, we used a well-controlled, double humanized BLT (dHu-BLT) mouse model harboring both a functional human immune system and a human-like gut microbiome to examine the effects of human FMT during suppressive ART. The objective of this study was to assess whether FMT could facilitate the restoration of gut microbial composition disrupted during suppressive ART, characterize host transcriptomic alterations following FMT, and evaluate the impact on systemic immune activation and inflammation. We anticipated that FMT would restore gut microbial composition, modulate gut transcriptional profile, and attenuate systemic inflammation and immune activation compared with non-FMT controls. For this, two groups of dHu-BLT mice, one supplemented with FMT in addition to ART (Rx+FMT) and a control group on ART alone (Rx), were used for the study. We longitudinally analyzed gut microbial composition, quantified the markers of systemic inflammation and immune activation in peripheral blood, measured markers of gut barrier damage and permeability, and profiled transcriptomic changes in GI tissue. Our results demonstrate that FMT in dHu-BLT mice is well-tolerated, does not induce aberrant immune activation or inflammation, reduces systemic inflammatory markers, and alters the expression of genes involved in cellular structure and organization, thereby suggesting a potential beneficial role of FMT for PLWH on ART.

## 2 Methods

### 2.1 Generation of double humanized BLT (dHu-BLT) mice

All the procedures used in this study involving animals complied with approved protocols by the Institutional Animal Care and Research Committee (IACUC) at the University of Nebraska-Lincoln (UNL). To generate double Hu-BLT (dHu-BLT) mice, first humanized bone marrow, liver, thymus (Hu-BLT) mice were generated according to the previously described method (25, 26). Briefly, 6–8 weeks old female irradiated (12 cGy/gm of body weight; RS200 X-ray irradiator, RAD Source Technologies, Inc, GA) NSG mice (NOD.Cg-PrkdcscidIl2rgtm1Wjl/SzJ; Cat# 005557; Jackson Laboratory, Bar Harbor, ME) were surgically implanted with a piece of human fetal thymic tissue fragment sandwiched between two pieces of human fetal liver tissue within the left renal capsule, followed by intravenous injection of 1.5-5 × 10^5^ CD34^+^ hematopoietic stem cells isolated from human fetal liver. An anesthesia cocktail of ketamine (0.1 mg/g) and xylazine (0.012 mg/g) in addition to analgesic buprenorphine (0.1 mg/kg) and antibiotic cefazolin (858 μg/mouse) was used during surgery. Human fetal thymus and liver tissue were procured from Advanced Bioscience Resources (Alameda, CA). Ten weeks after surgery, human immune reconstitution was measured in peripheral blood. Humanized BLT mice with similar reconstitution were then used to generate dHu-BLT mice following our previously reported method (27). Briefly, mice were first subjected to a broad-spectrum antibiotic cocktail consisting of ampicillin (1g/L), metronidazole (1g/L), neomycin (1g/L), and vancomycin (0.5g/L) for two weeks with daily cage change, followed by two oral gavages of 200 μL of mixed human fecal microbiota (Donor mix) 24 hours apart. Mixed human fecal microbiota suspension was prepared from an equal portion of 3 donor samples (2 males and 1 female, aged 26–29 years; OpenBiome, Massachusetts, US) in an anaerobic chamber.

### 2.2 Experimental approach

**Figure 1:**
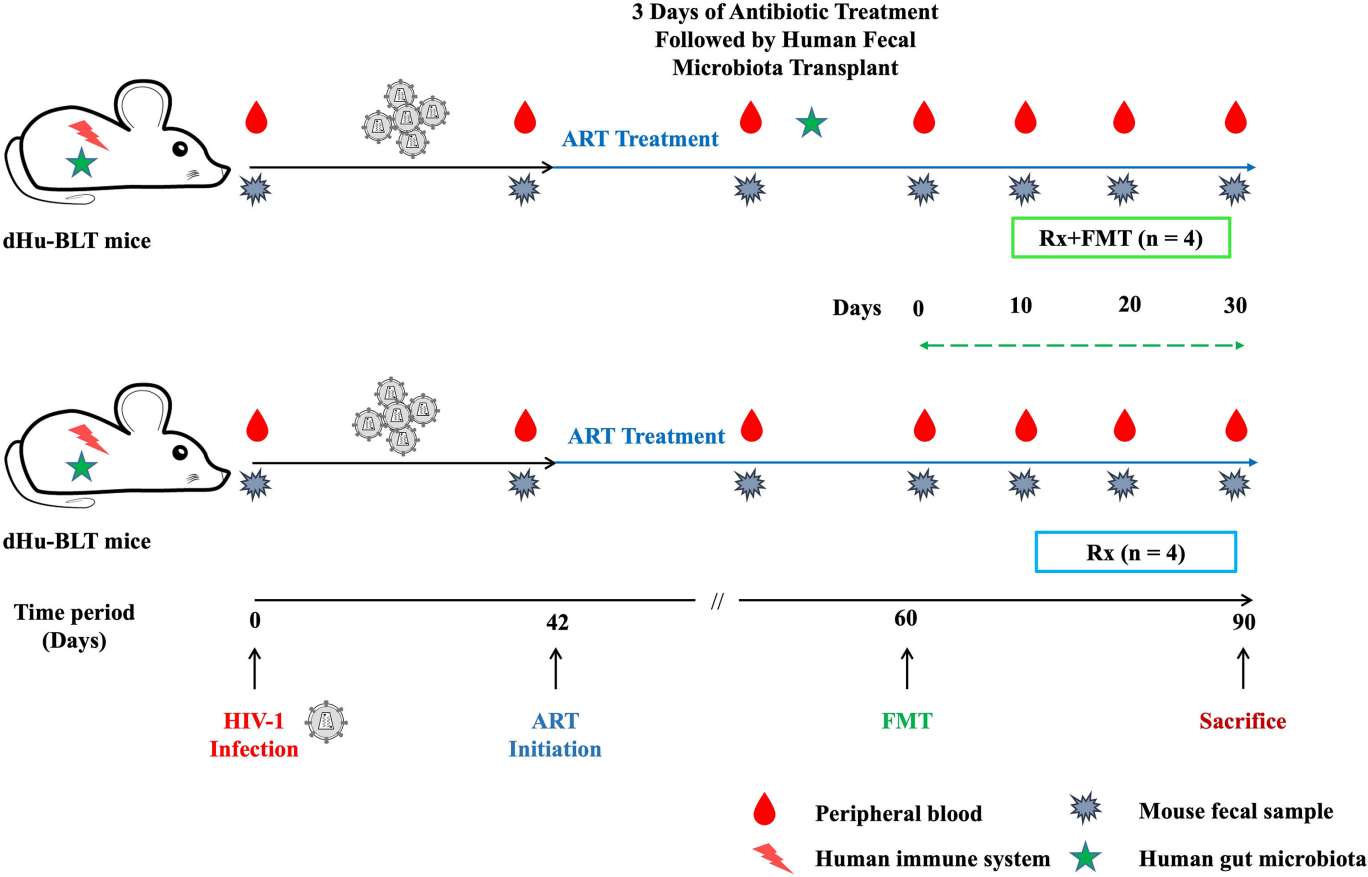
Two groups of dHu-BLT mice were infected with HIV. Following six weeks of infection, both groups received ART. Once plasma viral levels became undetectable, one group was inoculated with a healthy human fecal microbiota mixture. Fecal samples and plasma were collected at multiple time points, and colon tissue was harvested at the end of the experiment.

Two groups of dHu-BLT mice (n = 4 per group) were used for this study (**Figure 1**). Mice were fed with regular mouse chow (Teklad 2916; Inotiv, Madison, WI). Two weeks post-human fecal microbiota transplant, all the mice were intraperitoneally inoculated with 200 μL of 10^5^ tissue culture infectious dose 50 (TCID_50_) of HIV-1 SUMA and allowed to spread the HIV-1 infection. Six weeks post-infection, mice were treated with daily intraperitoneal injections of tenofovir disoproxil fumarate (TDF; 205 mg per kg bodyweight), emtricitabine (FTC; 211 mg per kg body weight), and dolutegravir (DTG; 56 mg per kg bodyweight). Two weeks after ART, once plasma viral load became undetectable, one of the treatment groups was subjected to a broad-spectrum antibiotic cocktail via drinking water for three days, with daily cage changes. This was followed by 24 hours of autoclaved drinking water and two oral gavages of 200 μL of Donor mix, administered 24 hours apart. A single FMT administration was employed to evaluate the effects of the initial (“founder”) microbial population capable of establishing persistent colonization and mediating sustained host responses, and to minimize procedure-related stress to host physiology due to the invasive oral gavage method. Four weeks after inoculating the human fecal microbiota (Donor mix), all the mice were euthanized using 100% carbon dioxide at a flow rate of 30-70% displacement of euthanasia chamber volume per minute. Fecal samples were collected at different time points for microbial analysis and fecal calprotectin measurement, along with peripheral blood for the evaluation of viral load, immune activation, and inflammatory markers. In addition, the distal section of the colon was collected for transcriptomic analysis. All the data were derived from a single independent experiment.

### 2.3 Human immune reconstitution and immunophenotyping

Human immune reconstitution and immune activation markers were measured in peripheral blood using a fluorescence-activated cell sorter (FACS) Aria II flow cytometer (BD Biosciences, San Jose, CA) and antibodies against mCD45-APC, hCD45-FITC, hCD19-PE/Cy5, hCD3-PE, hCD4-Alexa 700, hCD8a-APC-Cy7, hCD69-BV 785, hCD38-BV 421, and hHLA-DR-PE/Cy7 (catalog numbers 103111, 304006, 302210, 300408, 300526, 301016, 310932, 356618, and 307616 respectively; Biolegend, San Diego, CA). Raw data were analyzed with FlowJo (version 10.8.1; FlowJo LLC, Ashland, OR). All the hu-BLT mice used in this study have an average human immune reconstitution (hCD45^+^) of 70.04% (SD = 19.18) of the peripheral blood lymphocytes analyzed (**Supplementary Table 1**), similar to previously reported (28, 29).

### 2.4 Quantification of plasma viral loads

The plasma viral loads were measured using qRT-PCR as described previously (25). Briefly, QIAamp Viral RNA mini kit (Qiagen, Cat# 52906) was used to extract the viral RNA from the mouse plasma and AcroMetrix HIV-1 panel (ThermoFisher Scientific, Cat# 950470) following the manufacturer’s protocol and quantified using C1000 Thermal Cycler and the CFX96 Real-time system (Bio-Rad). The reaction mixture was set up using 5 μL of extracted viral RNA, TaqMan Fast Virus 1-Step master mix (ThermoFisher Scientific, Cat# 4444432), forward primer (5’-GCCTCAATAAAGCTTGCCTTGA-3’), reverse primer (5’- GGGCGCCACTGCTAGAGA-3), probe (/56-FAM/CCAGAGTCA/ZEN/CACAACAGACGGGCACA/3IABkFQ/) and top up to 20 μL with nuclease-free water.

### 2.5 Fecal sample collection and DNA extraction

Fecal samples were collected within the biosafety cabinet and were stored at -80 °C in an autoclaved Eppendorf tube until DNA extraction. Fecal DNA was extracted following the previously published method (30). Briefly, fecal samples were diluted in ice-cold phosphate-buffered saline (pH 7) (Cat# SH30256.02, Hyclone) in a 1:10 ratio and washed three times. The bacterial cells were then subjected to physical lysis using a bead beater (Mini-Beadbeater-16, Biospec) in presence of 0.1 mm zirconia/silica beads (Biospec) and phenol-chloroform-isoamyl alcohol (25:24:1) following incubation, first with lysis buffer [200 mM NaCl, 100 mM Tris (pH 8.0), 20 mM EDTA, 20 mg/ml lysozyme] at 37 °C and then with 10% sodium dodecyl sulfate solution and proteinase K (Cat# MC500B Promega) at 60 °C. After physical lysis, top layer was extracted twice with phenol-chloroform-isoamyl alcohol (25:24:1) followed by chloroform:isoamyl alcohol (24:1) two times. DNA was recovered by standard ethanol precipitation, which was resuspended in Tris-HCL buffer (10 mM, pH 8.0).

### 2.6 16S rRNA bacterial gene sequencing and analysis

16S ribosomal RNA (rRNA) bacterial gene sequencing was performed at the Genomics Core Facility of the University of Nebraska Medical Center. The hypervariable V3-V4 region of the bacterial 16S rRNA was sequenced using the Illumina MiSeq platform. The following primer sequences were used for the 16S library prep protocol: Forward Primer = 5′TCGTCGGCAGCGTCAGATGTGTATAAGAGACAGCCTACGGGNGGCWGCAG.

Reverse Primer = 5′GTCTCGTGGGCTCGGAGATGTGTATAAGAGACAGGACTACHVGGGTATCTAATCC.

Raw files received from the sequencing facility were processed following the DADA2 (v1.32) pipeline (31) at the University of Nebraska Holland Computer Center Swan cluster. A total of 9,232,988 reads were recovered after processing in DADA2. Taxonomy classification was performed in QIIME2 (32) using a self-trained naïve Bayes classifier based on SILVA SSU Ref NR 99 138.1 (33) for the V3-V4 region, prepared through RESCRIPt (34) pipeline. Unclassified taxa with a defined level of taxonomic resolution were renamed to their nearest classified higher taxon. The International Committee on Systematics of Prokaryotes (ICSP) proposed new names for the bacterial phylum were adopted (35). FastTree analysis (36) and MAFFT alignment (37) were used to generate a phylogenetic tree in QIIME2. Unassigned phyla and features assigned to the family of mitochondria, order of chloroplast, and singletons were removed from the analysis. A total of 8,852,924 reads (**Supplementary Figure 1**) and 1,754 ASVs were obtained after filtration, which was rarified to 90% of the minimum library size without replacement for downstream analysis using different R packages in R (version 4.4.1).

### 2.7 Total RNA extraction, sequencing, and processing

Total RNA was extracted from the distal section of the colon using RNeasy plus mini kit (Qiagen; Cat# 74134) according to the manufacturer’s protocol. Extracted total RNA was sent to CD Genomics (Shirley, NJ) for total RNA-seq. The quality of the sample was evaluated by Qubit RNA HS assay (ThermoFisher) and Bioanalyzer 2100 Eukaryote Total RNA Nano (Agilent Technologies, CA) and sequenced using the rRNA depletion method with NEBNext Ultra II (directional) and RiboZero Plus.

Raw reads were preprocessed using the command line tools Trimmomatic (38), i.e., each RNA-seq read was trimmed to make sure the average quality score was larger than 30 and had a minimum length of 70 bp. Sequences were mapped to the reference genomes using HISAT2 (39), allowing up to two base mismatches per read. Reads mapped to multiple locations were not considered. The number of reads in genes was counted by the software tool HTSeq-count (40) with the “union” resolution mode. DESeq2 was employed for differential analysis (41).

### 2.8 Plasma inflammatory markers measurement

Inflammation 20-Plex Human ProcartaPlex Panel (Cat# EPX200-12185-901, ThermoFisher Scientific, Waltham, MA), sCD163 Human ELISA Kit (Cat# EHCD163, ThermoFisher Scientific, Waltham, MA), sCD14 Human ELISA Kit (Cat# EHCD14, ThermoFisher Scientific, Waltham, MA), and Mouse FABP2 ELISA Kit PicoKine (Cat# EK1622, Pleasanton, CA) were used to measure inflammatory markers from plasma. Samples were run in duplicate following the manufacturer’s protocol. For the Inflammation 20-Plex Human ProcartaPlex Panel, Luminex MAGPIX instrument (Luminex Corporation, Austin, TX) was used to measure inflammatory markers according to the manufacturer’s protocol. Analytes out of range (OOR) in any sample were excluded from the statistical comparison.

### 2.9 Fecal calprotectin measurement

Fecal samples were first dissolved in 50X extraction buffer (0.1 M Tris, 0.15 M NaCl, 1.0 M urea, 10 mM CaCl2, 0.1 M citric acid monohydrate, 5 g/L BSA (pH 8.0)) and then centrifuged at 10,000 × g for 20 minutes at 4 °C. The supernatant was then used to measure the human calprotectin (S100A8/A9) level using Human S100A8/S100A9 Heterodimer DuoSet ELISA kit (DY8226-05, R&D systems, MN).

### 2.10 Statistical analysis

Welch’s t-test was performed if all data passed the Shapiro-Wilk test of normality; otherwise, a Wilcoxon rank sums test was performed to compare the statistical difference between groups. For beta diversity analysis, Permutational Multivariate Analysis of Variance (PERMANOVA), and Permutational Analysis of Multivariate Dispersion (PERMDISP) were used to assess statistical differences between groups with 999 randomizations. All analyses were performed in R (version 4.4.1).

## 3 Results

### 3.1 Successful engraftment of human donor fecal microbiota in dHu-BLT mice

**Figure 2:**
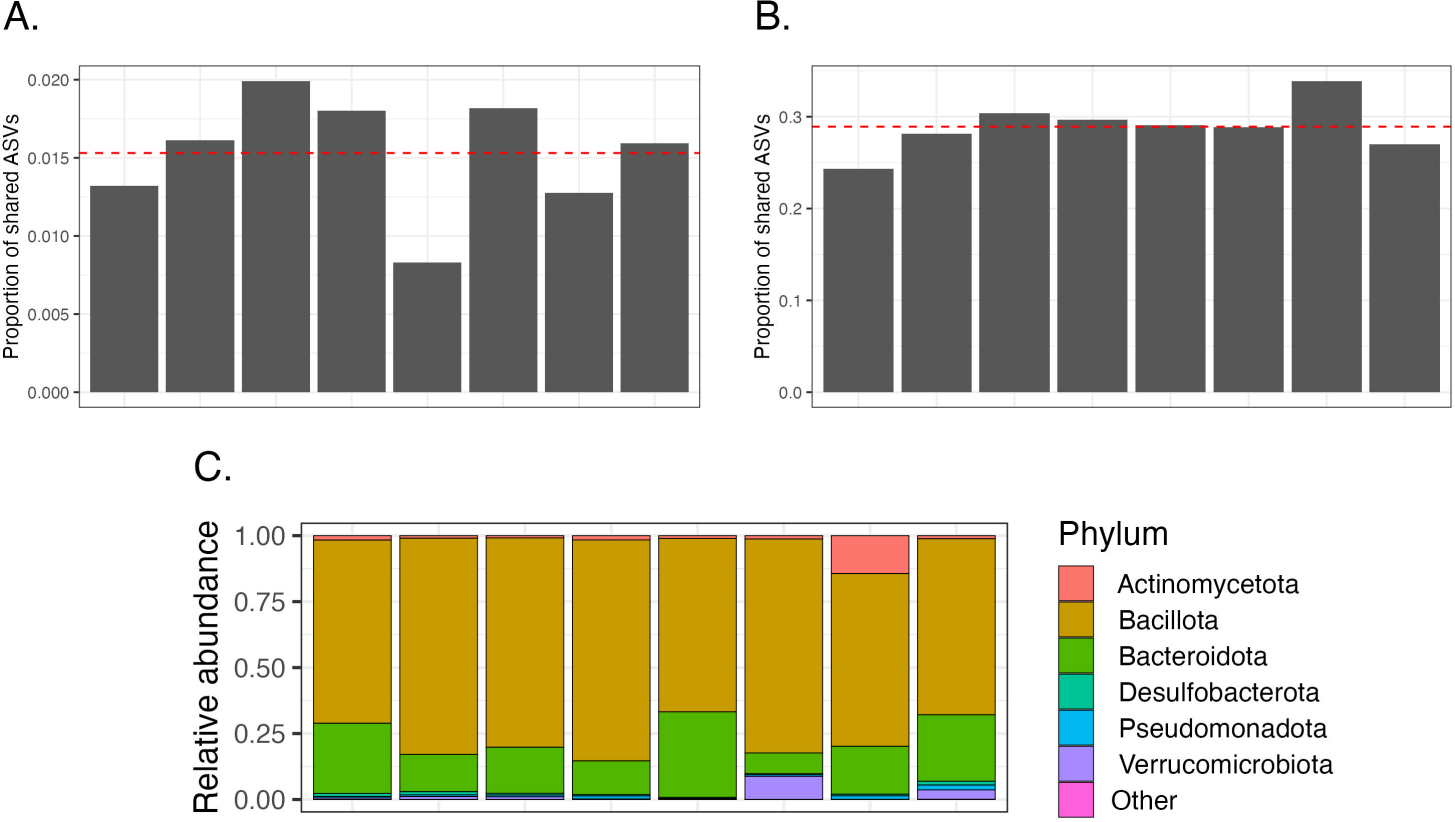
Engraftment of human donor fecal microbiome communities in dHu-BLT mice. The proportion of shared ASVs between the human donor fecal microbiota (Donor Mix) and humanized mice before antibiotic treatment **(A)**, two weeks after FMT **(B)**, and relative abundance at the phylum level at two weeks after FMT **(C)**. Each bar on the graph represents an individual mouse. The red dotted line represents the average shared ASVs. Phylum <0.005 abundance is grouped as “Other”.

Humanized-BLT mice were pretreated with a broad-spectrum antibiotic cocktail for 14 days prior to the transplantation of a human donor microbiota (Donor Mix, **Supplementary Figure 2**). This treatment resulted in a significant alteration of the murine gut microbiota, including an increased abundance of *Pseudomonadota* (formerly *Proteobacteria*) (**Supplementary Figure 3**), indicating a common post-antibiotic bloom of certain bacteria. Two weeks post-FMT, shared amplicon sequence variants (ASVs) between the donor mix and recipients were analyzed. Prior to human donor fecal microbiota engraftment, the average proportion of human donor mix ASVs was below 2%, while it was increased to an average of 28.92% following engraftment (**Figure 2**). Furthermore, 38 human donor-specific ASVs were detected in all dHu-BLT mice (**Supplementary Figure 4**; **Supplementary Table 2**). In addition, all major human bacterial taxa were successfully engrafted in our dHu-BLT mice (**Figure 2C**; **Supplementary Figure 5**).

### 3.2 Partial restoration of gut microbiome of HIV-infected dHu-BLT mice following FMT

**Figure 3:**
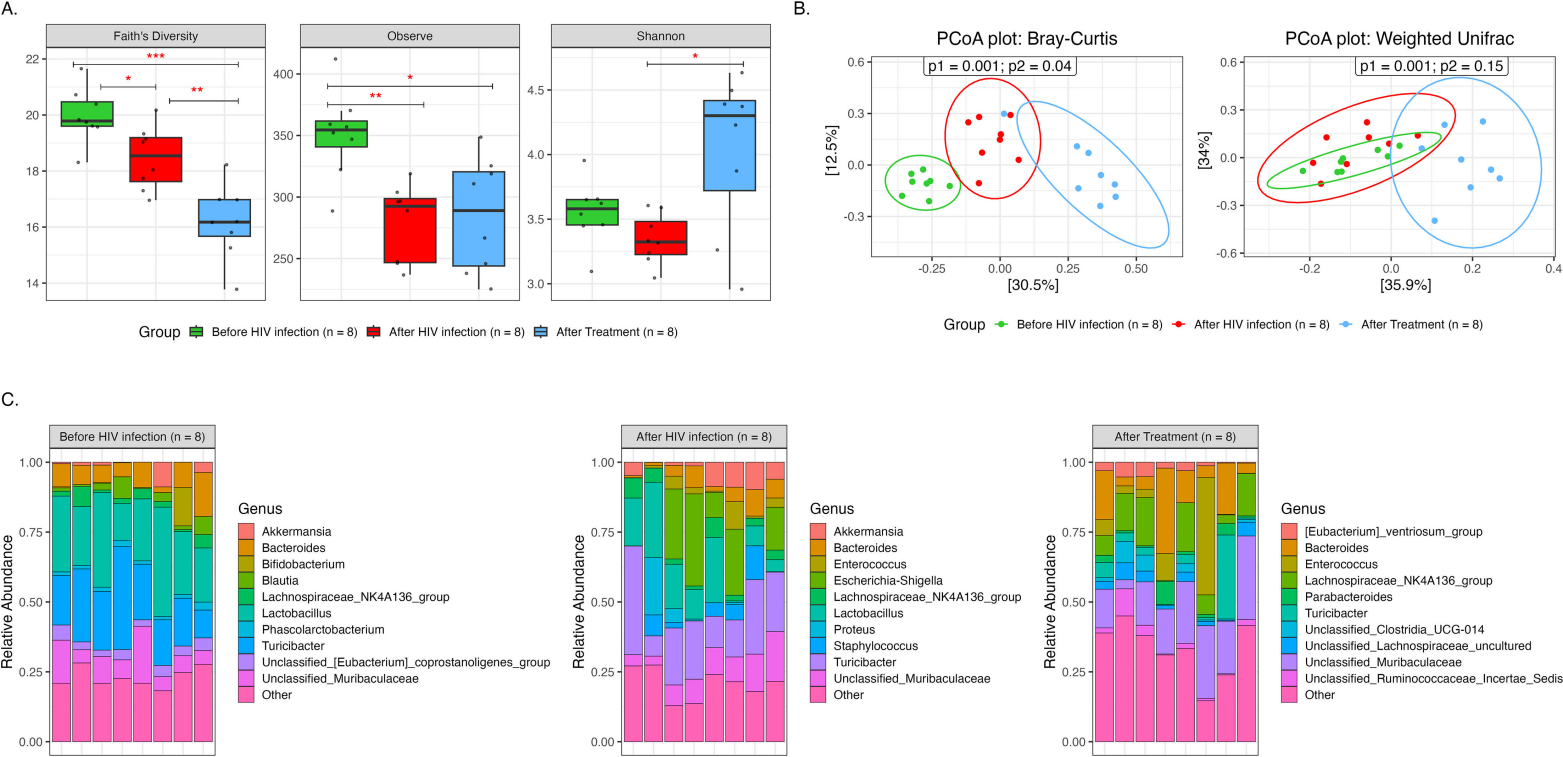
Shift in microbial diversity in dHu-BLT-mice following HIV infection and ART intervention. Alpha diversity **(A)**, beta diversity **(B)**, and relative abundance of the ten most abundant genera **(C)** before HIV infection, after HIV infection, and two weeks after ART. Each data point **(A, B)** and each bar **(C)** represent an individual mouse. Genera outside the ten most abundant were grouped as “Other.” p1 = Permutational Multivariate Analysis of Variance (PERMANOVA), and p2 = Permutational Analysis of Multivariate Dispersion (PERMDISP). *, **, and *** indicate significant differences with p < 0.05, p < 0.01, and p < 0.001, respectively, after the Tukey *post-hoc* test.

To evaluate the effects of HIV infection and ART on the engrafted gut microbiota in dHu-BLT mice, we analyzed the microbial diversity and composition before HIV infection, following the infection, and after ART administration. As expected, significant alterations in microbial composition were observed between these different conditions (**Figure 3**). Compared to the pre-HIV infection condition, both the observed number of species and the Faith’s diversity metric were significantly lower post-HIV infection and post-ART treatment. Similarly, Bray-Curtis dissimilarity and weighted UniFrac beta diversity metrics were significantly different between these conditions. In addition, several beneficial genera, including *Lactobacillus*, *Bifidobacterium*, and *Blautia*, that were reduced in abundance following HIV infection were not restored to the level before HIV infection. Nonetheless, potentially pathogenic genera such as *Escherichia-Shigella, Proteus*, and *Staphylococcus* that increased after HIV infection were reduced in abundance at two weeks after ART (**Figure 3C**). This finding indicates that ART alone was insufficient to fully restore microbial diversity and composition to the pre-infection levels.

### 3.3 FMT supplementation increased the relative abundance of beneficial bacteria

**Figure 4:**
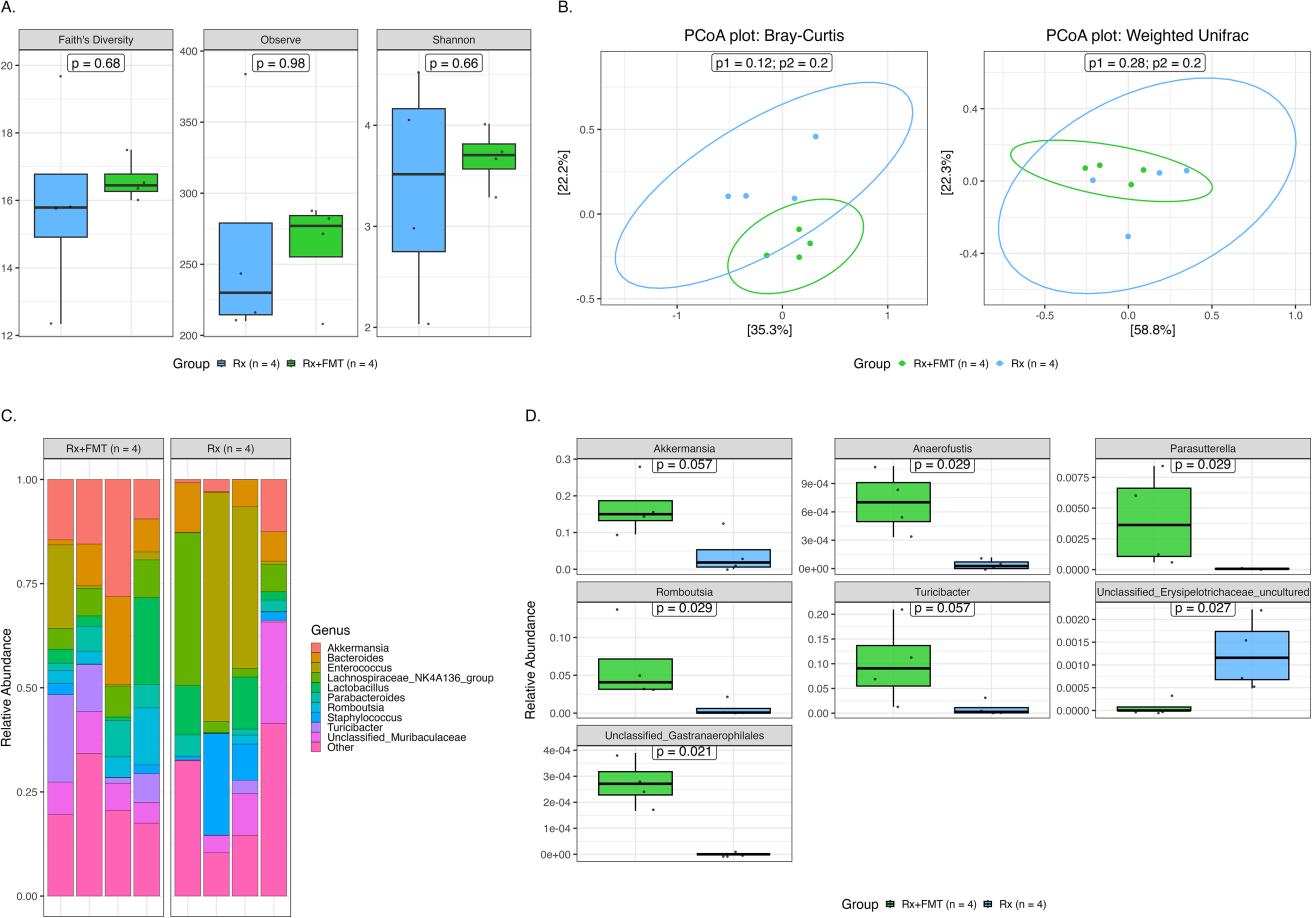
Microbial composition comparisons at 30 days post-FMT between ART plus FMT versus ART alone groups. Alpha diversity **(A)**, beta diversity **(B)**, relative abundance of the ten most abundant genera **(C)**, and the genera exhibiting the largest difference in the relative abundance at 30 days post-FMT **(D)**. Each data point in panels **(A, B, D)**, and each bar in panel **(C)**, represents an individual mouse. Genera outside the ten most abundant were grouped as “Other.” Significance (p-value < 0.05) was evaluated using the nonparametric Wilcoxon rank-sum test.

To assess the efficacy of FMT in restoring a healthy gut microbial community during suppressive ART, we administered healthy donor-derived fecal microbiota (Donor mix) to half of the ART-treated mice after peripheral viral load became undetectable in all the mice. Although no significant differences in microbial diversity indices were observed between mice receiving ART plus FMT compared to those receiving only ART at various time points (10, 20, and 30 days) (**Supplementary Figure 6**), there was a noticeable difference in the relative abundance of the different bacterial genera (**Figure 4C**). At 30 days after FMT (before mice were sacrificed to assess plasma inflammatory markers and gut tissue transcriptomic changes), the relative abundance of *Anaerofustis*, *Parasutterella*, and *Romboutsia* was significantly higher in the animals receiving ART plus FMT compared to those receiving only ART, while *Unclassified_Erysipelotrichaceae_uncultured* was reduced. Furthermore, two genera, *Akkermansia* and *Turicibacter*, were close to being significantly increased (p = 0.057) in the ART plus FMT group compared to the ART alone group (**Figure 4D**).

In addition, differential abundance (DA) analysis using ANCOM-BC2 (42), controlling for false discovery rate (FDR) at a threshold of q < 0.05 was used to detect differentially abundant genera. Although no genera shared between the ART plus FMT and ART alone groups were identified as differentially abundant by ANCOM-BC2, several genera were categorized as structured zeros (completely or nearly completely absent in one group) (**Supplementary Table 3**). Genera such as *Klebsiella*, *Anaeroplasma*, *Acetatifactor*, *Unclassified_Ruminococcaceae, Monoglobus, [Eubacterium]_nodatum_group, Unclassified_Erysipelotrichaceae_uncultured, Catabacter, Lachnospiraceae_ASF356, Lactiplantibacillus* were only present in the ART alone group, and genera such as *Christensenellaceae_R-7_group, Eisenbergiella,[Clostridium]_innocuum_group, Stenotrophomonas, Unclassified_Gastranaerophilales, Papillibacter, Acetanaerobacterium, Candidatus_Soleaferrea, Gordonibacter,Unclassified_Atopobiaceae_uncultured, Caproiciproducens, Unclassified_Desulfovibrionaceae_uncultured* were only present in mice supplemented with FMT.

### 3.4 FMT significantly altered the gut gene transcriptome

To evaluate the impact of FMT on mucosal gene expression during suppressive ART, we compared the transcriptomic alterations in the distal section of colon tissue from dHu-BLT mice treated with ART plus FMT versus ART alone. RNA reads were mapped to the reference human genome (GRCh38/hg38) and the mouse genome (GRCm38). A total of 19792 human genes and 29329 mouse genes were identified in this study.

**Figure 5:**
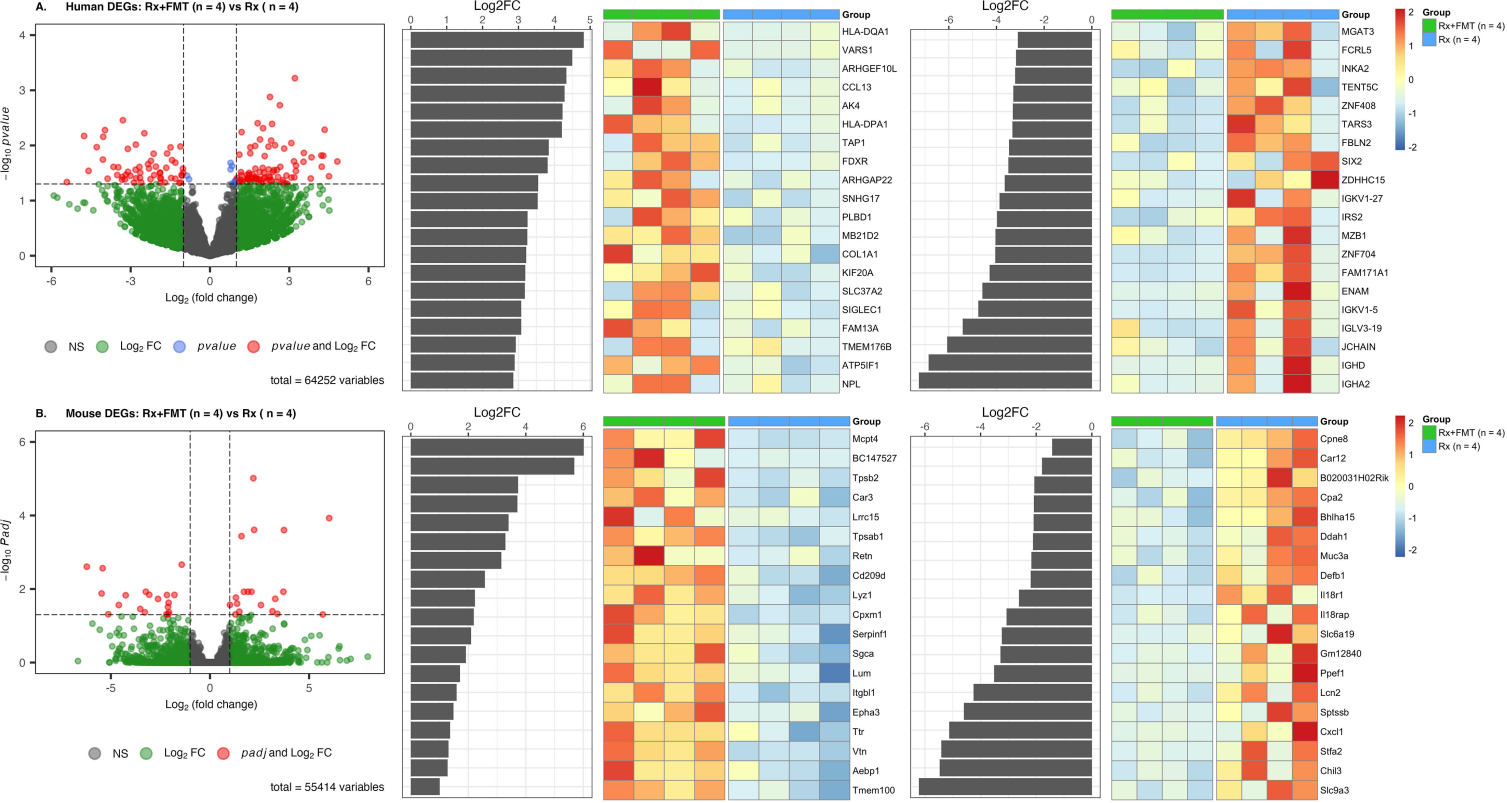
FMT alters the transcriptomes of both implanted human genes and endogenous murine genes in colon tissue. Volcano plots of differentially expressed human-related genes (p-value of <0.05 and |Log*_2_*FC| >1) **(A)** and murine genes (adjusted p-value of <0.05 and |Log*_2_*FC| >1) **(B)** along with a heatmap of the top 20 (human) and 19 (murine) upregulated or downregulated genes. Bar graphs show the Log*_2_* fold change of the top upregulated and downregulated transcripts.

First, we assessed the impact of FMT on the expression of human-related genes and evaluated the differentially expressed genes (DEGs). In total, 141 human DEGs (83 upregulated and 58 downregulated; p-value < 0.05 and | Log_2_FC | > 1) were identified (**Supplementary Table 4**). The top upregulated DEGs included major histocompatibility complex, class II, DQ alpha 1 (*HLA-DQA1*), valyl-tRNA synthetase 1 (*VARS1*), rho guanine nucleotide exchange factor 10 like (*ARHGEF10L*), C-C motif chemokine ligand 13 (*CCL-13*), and adenylate kinase 4 (*AK4*), with *HLA-DQA1* being the most upregulated gene (Log_2_FC = 4.82). The most downregulated genes were immunoglobulin heavy constant alpha 2 (*IGHA2*), immunoglobulin heavy constant delta (*IGHDI*), joining chain of multimeric IgA and IgM (*JCHAIN*), immunoglobulin lambda variable 3-19 (*IGLV3-19*), and immunoglobulin kappa variable 1-5 (*IGKV1-5*), with *IGHA2* being the most downregulated gene (Log_2_FC = -7.25) (**Figure 5A**).

Regarding endogenous murine gene expression patterns, a total of 38 DEGs (19 upregulated and 19 downregulated; FDR-adjusted p-value < 0.05 and |Log_2_FC| > 1) were identified in total (**Supplementary Table 5**). The top upregulated DEGs in this comparison included mast cell protease 4 (*Mcpt4*), cDNA sequence BC147527 (BC147527), tryptase beta 2 (*Tpsb2*), carbonic anhydrase 3 (*Car3*), and leucine-rich repeat containing 15 (*Lrrc15*), with *Mcpt4* being the most upregulated gene (Log_2_FC = 6.01). The most downregulated genes were solute carrier family 9, member A3 (*Slc9a3*), chitinase-like 3 (*Chil3*), stefin A2 (*Stfa2*), chemokine (C-X-C motif) ligand 1 (*Cxcl1*), and serine palmitoyltransferase (*Sptssb*), with *Slc9a3* being the most downregulated gene (Log_2_FC = -6.21) (**Figure 5B**).

**Figure 6:**
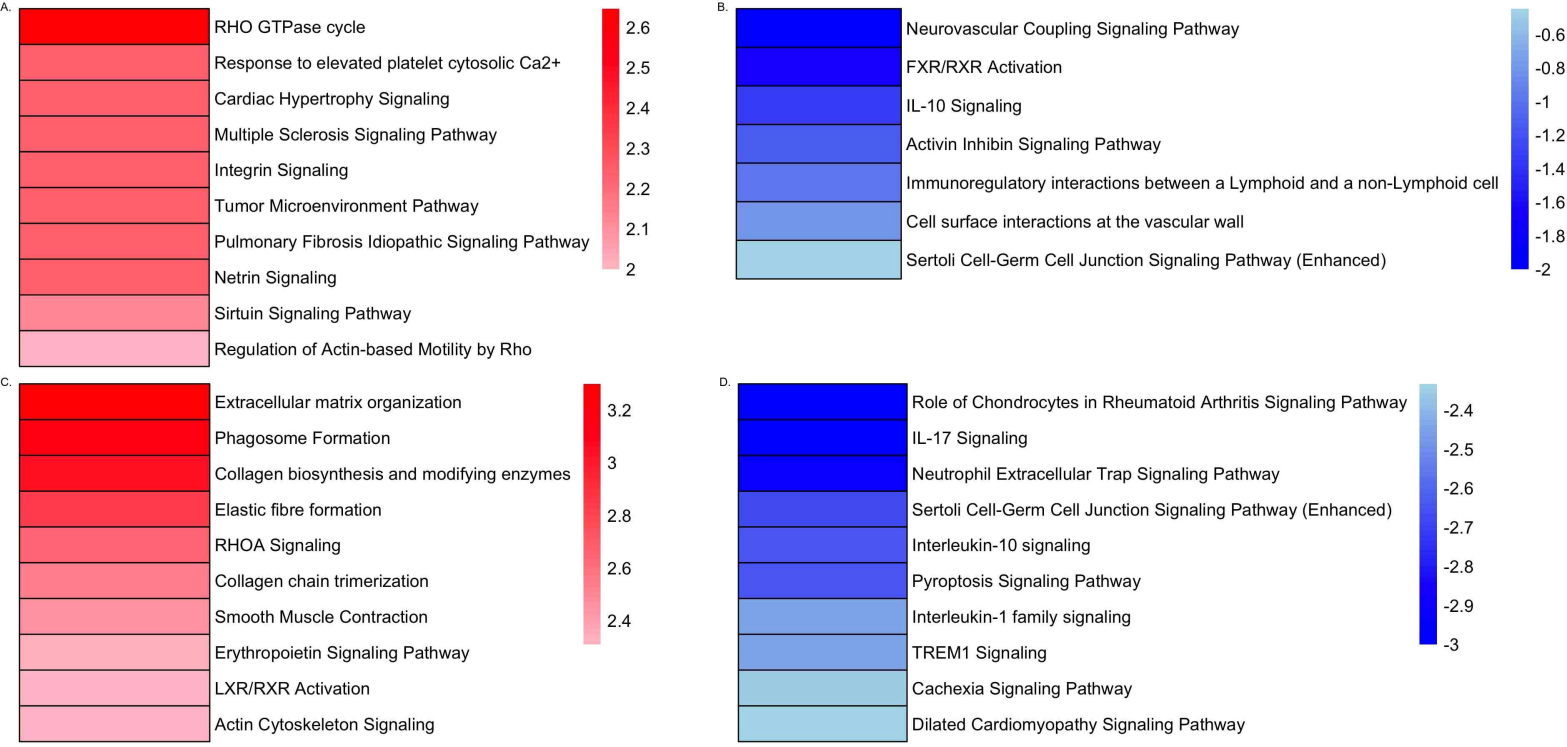
Altered canonical signaling pathways in the colon tissue at 30 days after FMT in the ART plus FMT group compared to the ART alone group. Panels **(A)** and **(B)** represent the top strongly activated and inactivated human canonical pathways, while panels **(C)** and **(D)** represent the top strongly activated and inactivated mouse canonical pathways. Pathways are color-coded by Z-score: red or lighter shades of red indicate a positive Z-score (activation), and blue or lighter shades of blue indicate a negative Z-score (inactivation). The top ten most strongly activated and inactivated pathways are shown in the image.

To better understand the biological processes broadly affected by FMT during suppressive ART, Ingenuity Pathway Analysis (IPA) with a gene expression threshold of absolute Log_2_FC >1 and p-value < 0.05 was used to identify the canonical cellular pathways altered by FMT treatment. Although the top ten activated pathways do not include major human immune function or inflammation-related pathways, IL-10 signaling and immunoregulatory interactions between a lymphoid and a non-lymphoid cell were among the top ten inactivated pathways (**Figure 6B**; **Supplementary Table 6**).

In the context of murine-related pathways, most of the activated pathways were related to cellular and structural organizations such as extracellular matrix organization, collagen biosynthesis and modifying enzymes, elastic fiber formation, collagen chain trimerization, smooth muscle contraction, and actin cytoskeleton signaling (**Figure 6C**; **Supplementary Table 7**). Inactivated pathways included those related to inflammation and immunity, such as IL-17 signaling, neutrophil extracellular trap signaling, interleukin-10 signaling, pyroptosis signaling, and interleukin-1 family signaling (**Figure 6D**; **Supplementary Table 7**).

### 3.5 FMT lowered plasma inflammatory markers but failed to reduce immune activation during suppressive ART

**Figure 7:**
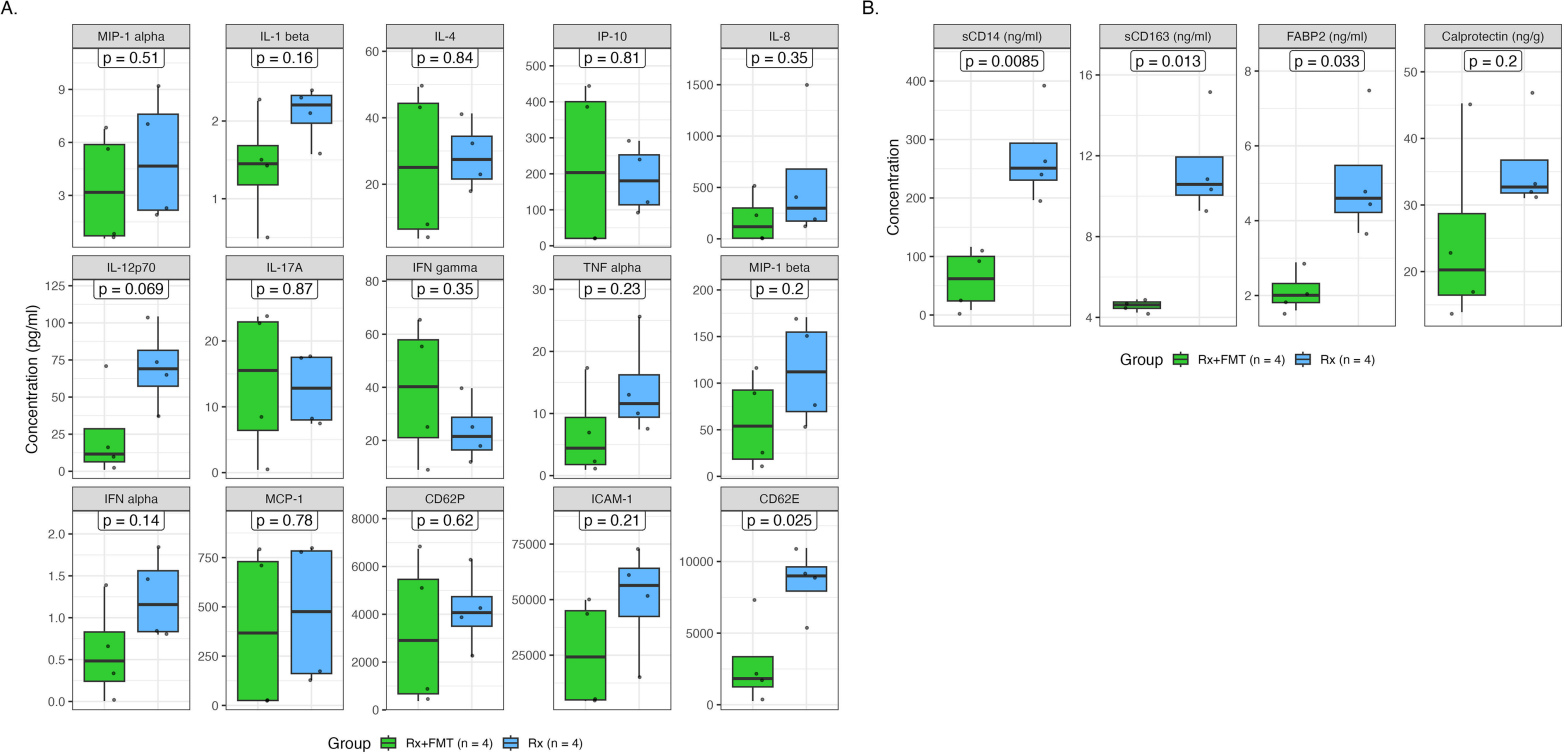
FMT lowered several plasma inflammatory markers **(A)** and markers of gut barrier integrity and inflammation **(B)** during suppressive ART. Each data point represents an individual mouse. Significance (p-value < 0.05) was evaluated using either the Welch’s t-test or the Wilcoxon rank sum test.

To evaluate the impact of FMT on systemic inflammation and immune activation, we monitored immune activation at multiple time points and assessed various inflammatory markers (cytokines, chemokines, cell adhesion, and inflammatory molecules) at the end of the experiment. Despite the lack of significant changes in peripheral blood immune population and reduction in T lymphocyte activation markers (CD38, CD69, and HLA-DR on both CD4^+^ and CD8^+^ T cells) (**Supplementary Table 8**), a notable decrease in specific plasma inflammatory markers was detected (**Supplementary Table 9**). Compared to the ART alone group, animals in the ART plus FMT group exhibited significantly lower plasma levels of soluble CD62E, a marker of endothelial cell activation and inflammation (43) (**Figure 7A**). Additionally, other inflammatory markers, particularly markers of gut barrier integrity and permeability (sCD14, sCD163, and FABP2) in plasma samples, and markers of intestinal inflammation (fecal calprotectin) in the fecal samples were quantified. Relative to the ART alone group, mice in ART plus FMT demonstrated a significant reduction in plasma levels of sCD14, sCD163, and FABP2 (**Figure 7B**). However, no significant differences in fecal calprotectin levels were observed between the groups.

## 4 Discussion

Although ART effectively suppresses HIV infection, it fails to restore gut microbial homeostasis or prevent microbial translocation, a key driver of systemic persistent immune activation and inflammation (1–4). Consequently, PLWH remain at heightened risk for HIV-associated comorbidities, highlighting the need for interventions that target microbial dysbiosis during suppressive ART (8). In this study, we therefore investigated the impact of FMT during suppressive ART using a well-controlled animal model. We longitudinally characterized the gut microbiome before HIV infection, after infection, and following ART administration. Finally, we assessed the potential of FMT as an adjunctive therapy by comparing microbial composition, gut transcriptomic profiles, and immunological and inflammatory markers between mice receiving ART plus FMT versus ART alone.

HIV exerts substantial effects on both the immune system and the gut microbiota (1, 12, 13). However, most studies have relied solely on human immune-reconstituted mice, overlooking the human microbial component. To better recapitulate the characteristics of HIV infection in humans within a mouse model, we first preconditioned the mouse’s gut with broad-spectrum antibiotics to create a niche for subsequent engraftment of donor microbes, a practice commonly utilized before FMT (44). We then generated the double humanized-BLT mice (dHu-BLT) by engrafting hu-BLT mice with a pooled fecal microbiota derived from three healthy donors. Although the proportion of engrafted human microbiota in our study was relatively lower compared to the engraftment proportion in the clinical setting, which could be due to anatomical and physiological differences between the mouse and human gastrointestinal tract, it was similar to the proportion previously reported in the dHu-BLT mouse model (29, 45). Importantly, donor microbiota engraftment in the recipient is often incomplete and heterogeneous, and functional effects are thought to depend primarily on the establishment and expansion of key microbial taxa rather than complete donor microbiota replacement (46, 47). Notably, the dHu-BLT model utilized in our study predominantly consists of the major human bacterial phyla, specifically *Bacillota* (formerly *Firmicutes*) and *Bacteroidota*, which together constitute over 90% of the total human gut microbial community (48). Additionally, other less dominant phyla, such as *Pseudomonadota* (formerly *Proteobacteria*), *Actinomycetota* (formerly *Actinobacteria*), and *Verrucomicrobiota* (48) were also successfully engrafted (**Figure 2**).

Two weeks after the generation of dHu-BLT mice, we infected all dHu-BLT mice with transmitted/founder HIV, SUMA. As anticipated, HIV infection leads to significant alterations to the gut microbiota. Several studies have reported a substantial decline in alpha diversity following HIV infection (11, 49, 50). Consistent with these previous findings, in our study, all the alpha diversity metrics evaluated were reduced after HIV infection, including a significant reduction in the observed number of species and Faith’s diversity compared to the status before infection (**Figure 3A**). In addition, HIV infection markedly altered different genera in our study. Notably, there was a marked reduction in the relative abundance of the beneficial genera like *Lactobacillus*, *Bifidobacterium*, and *Blautia*, accompanied by an increase in the relative abundance of potentially pathogenic genera, such as *Escherichia-Shigella*, *Proteus*, and *Staphylococcus* (**Figure 3C**). Similar shift in microbial composition, characterized by enrichment of potentially pathogenic bacteria and depletion of beneficial bacteria in HIV infected individuals, has been reported in other studies (11). Furthermore, we also compared gut microbial alteration following ART. Some studies suggested the potential of ART to correct HIV-associated microbial dysbiosis with partial restoration of gut microbiota and an increase in diversity compared to before ART (50, 51). Although an increase in Shannon diversity was observed, microbial diversity did not recover to the levels before HIV infection. However, the relative abundance of potentially pathogenic genera, such as *Escherichia-Shigella*, *Proteus*, and *Staphylococcus*, that were increased after HIV infection was reduced following ART treatment (**Figure 3C**). These observations indicate that ART alone was insufficient to fully restore microbial diversity and composition to the pre-infection levels, highlighting the need for adjunctive therapeutic strategies like transplantation of fecal microbiota from a healthy donor.

FMT is an emerging therapeutic intervention that replenishes gut microbial diversity. Several clinical studies demonstrated the beneficial effects of FMT in gastrointestinal disorders and potential applications beyond the GI tract (14–16, 19–21). To explore the potential of FMT as an adjunct therapy in PLWH on ART to restore gut microbial diversity, enhance mucosal barrier function, and mitigate residual immune activation and inflammation, we administered FMT from healthy human donors (Donor Mix) to half of the ART-treated mice once the plasma viral load became undetectable (**Supplementary Table 10**). Although microbial diversity did not differ significantly between the groups (**Supplementary Figure 6**), FMT treatment enriched several bacterial genera linked to metabolic and gut health (**Figure 4D**). For instance, *Parasutterella*, a genus associated with bile acid metabolism and low-density lipoprotein regulation (52, 53), *Anaerofustis*, a known producer of short-chain fatty acids such as acetate and butyrate (54), and *Romboutsia*, previously reported to improve gut endothelial function and expression of tight junction protein (Claudin5) in high-fat diet-fed mice (55), were significantly increased in our FMT-supplemented group compared to the ART alone group. In addition, although statistically not significant (p = 0.057), it is important to note the increased abundance of *Akkermansia* in the FMT-supplemented group. *Akkermansia*, a mucin-degrading bacterium, has been extensively linked to its ability to improve gut health through multiple mechanisms, including regulation of tight junction proteins (56), reinforcement of enterocyte monolayer integrity (57), and enhancement of intestinal stem cell proliferation and regeneration (58). In PLWH, a decreased abundance of *Akkermansia* has been linked to metabolic disorders (59), whereas its presence has been shown to mitigate HIV-1 gp120-induced neuronal pyroptosis (60). The beneficial effects of FMT in our study might have been through an increase in the abundance of some of these beneficial bacteria, including *Akkermansia.* Moreover, ANCOMBC2 analysis also reveals several genera uniquely present in each group. Such alteration of microbial composition, particularly those associated with maintaining gut integrity, might play a crucial role in preventing microbial translocation, thereby helping to control unwanted systemic immune activation and inflammation in HIV-infected individuals receiving ART.

FMT is known to modulate the host gene expression, considerably influencing gut physiology (17–20). In the current study, we also examined the associated transcriptomic alteration in the distal section of colon tissue. The transcriptomic profile of human-related genes suggests the potential of FMT to modulate the immune system towards active surveillance. The significant upregulation of antigen processing and presentation genes, such as *HLA-DQA1* and *HLA-DPA1*, suggests enhanced overall antigen surveillance, including towards the new microbial community engrafted through FMT. This is further supported by the elevation of *TAP1*, a key transporter associated with the MHC processing machinery (61). Conversely, immunoglobulin-related genes (*IGLV3-19, IGLV1-5, IGKV1-27, IGHA2, IGHD, JCHAIN, FCRL5*) were downregulated, likely reflecting a restoration of gut-microbial homeostasis and reduced inflammatory signaling, resulting in limited plasma-cell differentiation and immunoglobulin gene transcription required to contain leaky gut or neutralize pathobionts. A similar result of decreased immunoglobulin production following FMT was reported previously in the dextran sulfate sodium- induced colitis mouse model (62). Collectively, these findings suggest that engrafted fecal microbiota may facilitate mucosal immune modulation. However, our study is limited to global transcriptional changes in the gut tissue. The direct impact on gut immune cells requires further investigation using a targeted approach, such as immunophenotyping or spatial transcriptomics of gut tissue at single-cell resolution, which was not performed in this study. Among the endogenous murine genes (**Figure 5B**), the largest upregulation was observed in *Mcpt4*, a gene associated with the regulation of intestinal epithelial migration and barrier function (63). In addition, genes such as *Tpsb2, Tpsab1, Lum*, and *Car3* involved in tissue maintenance and protection were also upregulated. *Tpsb2* and *Tpsab1* encode tetramer-forming tryptases (MCP-6 and MCP7), which enable heparin binding activity and peptidase activity (64), while *Lum* encodes protein Lumican, known to regulate collagen fibril assembly (65). *Car3* is associated with the protection of cells from oxidative damage in the liver and skeletal muscle (66). Furthermore, genes such as Leucine-rich repeat-containing protein 15 (*LRRC15*) and Integrin beta-like 1 (*ITGBL1*) are related to ECM interaction and adhesion and binding activity (67, 68) were also upregulated. Conversely, some of the downregulated genes were related to an inflammatory response. *IL18r1* and *IL18rap*, the essential components for IL18-mediated signal transduction, were downregulated. Furthermore, *CXCL1*, promoter of proinflammatory reactions (69), and *Lcn2*, a gene encoding the acute-phase protein Lipocalin-2 that promotes intestinal epithelial cells’ pyroptosis (70), were downregulated. Collectively, these transcriptomic changes suggest that FMT may enhance mucosal repair and attenuate inflammation, a process critically impaired in HIV infected individuals.

To understand the biological process associated with DEGs, IPA was performed. In the context of human immune-related pathways, several pathways were either activated or inactivated (**Figures 6A, B**). Notably, IL-10 was one of the human-immune-related pathways that was inactivated. IL-10 is an important anti-inflammatory cytokine that suppresses immune cell activity (71). *In vitro* analysis suggests an inhibitory role of IL-10 in CD4^+^ and CD8^+^ T cell proliferation and cytokine production (72, 73). Several studies have suggested the pathogenic role of IL-10 in HIV infection, with elevated IL-10 levels associated with viral load and disease progression (72–74). Among the murine-related pathways analyzed, ECM organization exhibited the strongest activation, accompanied by collagen biosynthesis and modifying enzymes, elastic fiber formation, and collagen chain trimerization, all of which collectively support gut structural integrity (**Figure 6C**). For instance, collagens provide tensile strength and are involved in cellular processes such as cell adhesion, migration, and tissue development (75), whereas Elastin supports low stiffness, high extension, and efficient elastic-energy storage function (76). Furthermore, smooth muscle contraction and actin cytoskeleton signaling were among the other top ten structure-related pathways that were activated. This observation is particularly relevant to HIV infection, which is known to induce extensive intestinal mucosa damage, including injury to GALT, disruption of the tight junction proteins, enterocyte apoptosis, and cellular cytoskeleton alteration (77, 78). Importantly, this gut injury persists despite effective viral suppression with ART (78). In contrast, the majority of the top ten downregulated pathways include pathways related to inflammation (**Figure 6D**), including multiple proinflammatory and interleukin-related pathways such as interleukin-17 signaling and interleukin-1 family signaling. In addition, pyroptosis signaling, which is an inflammatory programmed cell death pathway (79), was also deactivated. Collectively, this coordinated shift in the murine transcriptome, specifically the downregulation of inflammatory pathways and upregulation of pathways related to cellular structure and organization, suggests a transition from an inflammatory state toward a tissue repair and structural remodeling program. These findings further imply that reshaping the microbial community through FMT may confer beneficial effects on the intestinal microenvironment in PLWH.

In addition to the ability of FMT to restore the gut microbiota and modulate gene expression, we also assessed its potential impact on systemic immune activation and inflammation. Despite achieving viral suppression, aberrant immune activation and inflammation represent significant challenges for PLWH on ART (7). Although the precise mechanisms driving these immune responses and inflammation remain unclear, dysregulation of the gut microbiota and microbial translocation have been strongly associated with such detrimental conditions (4–6). Numerous studies have demonstrated the potential of FMT to mitigate inflammation locally and systemically (20–22, 80). Thus, we longitudinally monitored markers of T cell activation at different time points after FMT. Despite the lack of a significant reduction in immune activation following FMT in the ART plus FMT group compared to the group receiving ART only, the absence of aberrant T cell activation post-FMT suggests that FMT was well tolerated. Further, several plasma inflammatory markers were assessed at the end of the study (**Figure 7A** and B). Compared to the ART alone group, the level of CD62E was significantly lower in the ART plus FMT group. CD62E, a leukocyte adhesion molecule, is strongly upregulated by inflammatory cytokines, such as TNF-α and IL-1β (43). Elevated CD62E has been reported in PLWH on long-term suppressive ART (81), potentially indicating prolonged exposure to ART resulting in endothelial damage. Lower levels of CD62E in the FMT-supplemented group in our study may suggest the ability of FMT to correct endothelial damage in HIV infection while on ART. In addition, we also evaluated markers of gut barrier damage and permeability (sCD14, sCD163, and FABP2). In a healthy state, the gut barrier functions as a critical defense mechanism to prevent the translocation of microorganisms and microbial products, such as lipopolysaccharides, into systemic circulation (82). However, HIV infection breaches the gut barrier integrity, resulting in increased permeability, leaky gut, and a subsequent increase in translocation of microbial products (82). Despite the early ART initiation, microbial translocation persists, indicating continuous enterocyte damage and ongoing bacterial translocation (83). In our study, markers of microbial translocation, innate immune activation, and barrier damage (sCD14, sCD163, and FABP2) were significantly lower in the ART plus FMT group compared to the ART alone group. These significant reductions of markers of innate immune activation (sCD14 and sCD163) in the absence of corresponding significant decreases in T-cell activation markers (CD38, CD69, and HLA-DR) suggest that the immunologic effects of FMT in this study primarily involved the gut–innate immune axis. Restoration of intestinal barrier integrity following FMT may reduce microbial translocation and subsequent monocyte activation, representing an early innate immune response to microbiome restructuring. In contrast, T-cell activation is influenced by multiple factors, including persistent antigen exposure, inflammatory cytokine signaling, and residual viral reservoirs (84, 85). A single FMT intervention with only four weeks of follow-up in our study may be insufficient to produce detectable changes in the adaptive immune compartment.

Although our study demonstrated a beneficial role of FMT during suppressive ART, it is important to acknowledge some of the limitations of our study. Our findings show that FMT-induced alterations in the gut microbiome were accompanied by host transcriptomic changes and reduced inflammation; however, these findings are observational and do not establish a causal mechanistic relationship. Future studies, including secondary fecal microbiota transplantation from responder mice into naïve recipients, will be required to determine whether the beneficial phenotype is transferable. Additionally, complete engraftment of human gut microbiota could not be achieved in our mouse model. Certain beneficial microbes that require a human-specific gut environment for successful colonization may have been absent, potentially influencing the outcome observed in our study. Furthermore, only a single FMT administration was performed; repeated FMT intervention might further enhance the observed beneficial effects. Nevertheless, we still detected a considerable beneficial impact of FMT in improving gut health and mitigating systemic inflammation during suppressive ART.

## 5 Conclusion

In summary, our findings indicate that healthy human FMT administered to ART-suppressed, HIV-infected dHu-BLT mice increases the relative abundance of beneficial bacteria, induces significant transcriptomic alterations in the gut tissue, and reduces inflammatory markers. These outcomes highlighted the positive impact of FMT on gut mucosal integrity and the regulation of systemic inflammation. Furthermore, no evidence of aberrant immune activation or increased inflammation was observed post-FMT, indicating that FMT intervention was well tolerated in our preclinical mouse model and may serve as a potential adjunctive strategy warranting further investigation in PLWH in the clinical setting.

## Data Availability

The datasets generated during the current study are available in the NCBI Sequence Read Archive (SRA) repository with accession number PRJNA1168946. Scripts related to the current study are freely available from the corresponding author upon request.
