# Supplementary material for: Fecal microbiota transplantation reduces inflammation and modulates gene expression in HIV-infected double humanized-BLT (dHu-BLT) mice on antiretroviral therapy
Source: Front Immunol. 2026 Jun 3;17:1773716. doi: 10.3389/fimmu.2026.1773716 (PMC13272091; doi:10.3389/fimmu.2026.1773716)
Supplement: Supplementary file 1 [file DataSheet1.docx]

Title: Fecal microbiota transplantation reduces inflammation and modulates gene expression in HIV-Infected double humanized-BLT (dHu-BLT) mice on antiretroviral therapy

Authors: Saroj Chandra Lohani^1,2, 3^, Chi Zhang^3^, Subhra Mandal^2,3†^, Miaoyun Zhao^1,2,3^, Yilun Cheng^2,3^, Amanda E. Ramer-Tait^4,5^, Qingsheng Li^1,2,3*^

Authors’ affiliations:

^1^HIV Cure and Viral Diseases Center, The Wistar Institute, Philadelphia, PA, United States

^2^Nebraska Center for Virology, University of Nebraska-Lincoln, Lincoln, NE, United States

^3^School of Biological Sciences, University of Nebraska-Lincoln, Lincoln, NE, United States

^4^Department of Food Science and Technology, University of Nebraska-Lincoln, Lincoln, NE, United States

^5^Nebraska Food for Health Center, University of Nebraska-Lincoln, Lincoln, NE, United States

† Present address: Corner Therapeutics, Watertown, MA, United States

* Address correspondence to Qingsheng Li, [qli@wistar.org](mailto:qli@wistar.org)

**Supplementary figures**


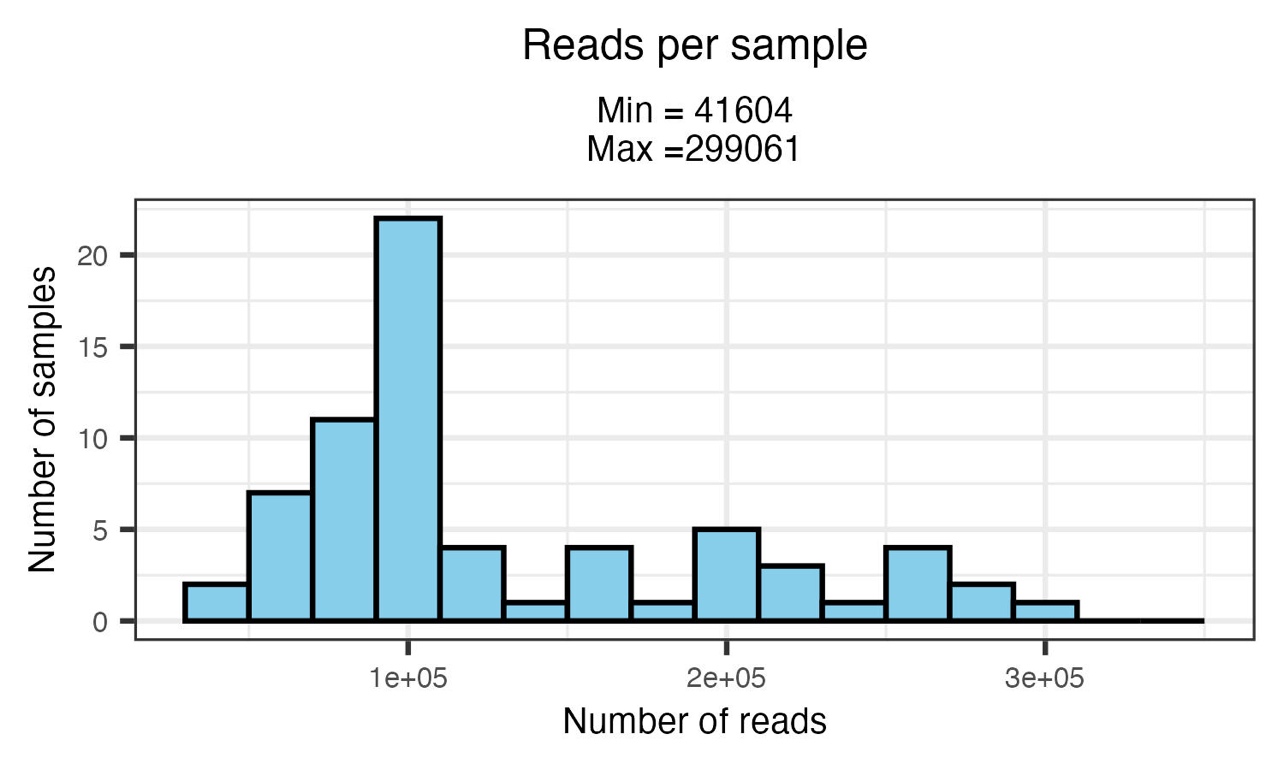


Figure S1: Read count distribution of dHu-BLT mouse fecal samples after filtering out unassigned features at the phylum level, features assigned to the family of mitochondria, order of chloroplast, and singletons.


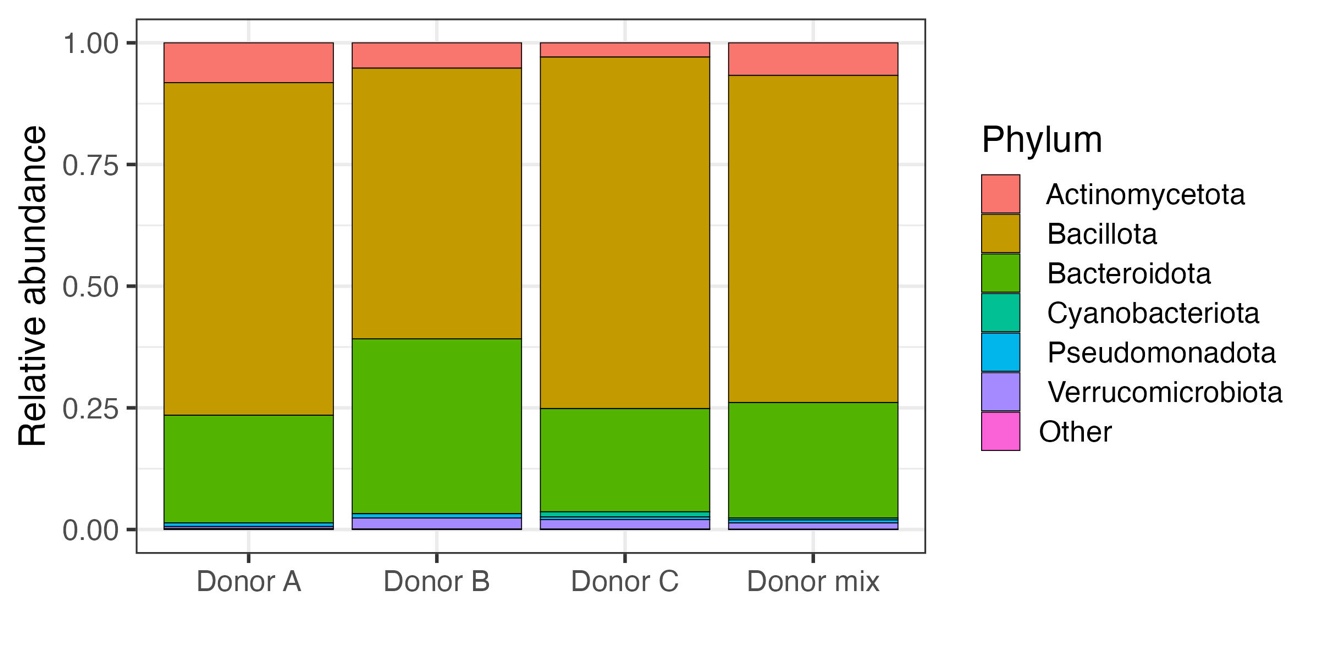


Figure S2: Microbial composition of human donor samples used for FMT. The figure illustrates the relative abundance of different phyla in each donor sample and donor mix. Each bar on the graph represents an individual donor or donor mix. Phylum < 0.005 abundance is grouped as "Other".


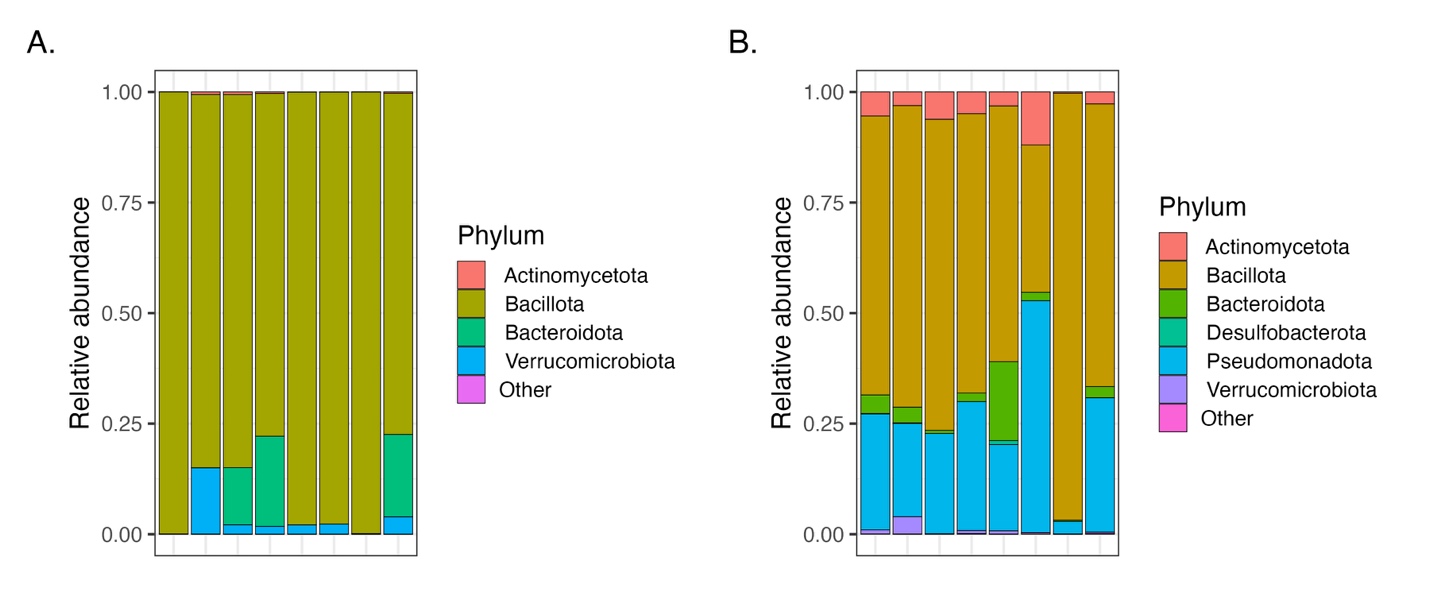


Figure S3: The gut microbiota composition of BLT mice included in the study. The figure displays the relative abundance of various phyla in each mouse prior to antibiotic treatment (A) and 14 days post-antibiotic-treatment (B). Each bar on the graph represents an individual mouse. Phylum < 0.005 abundance is grouped as "Other".


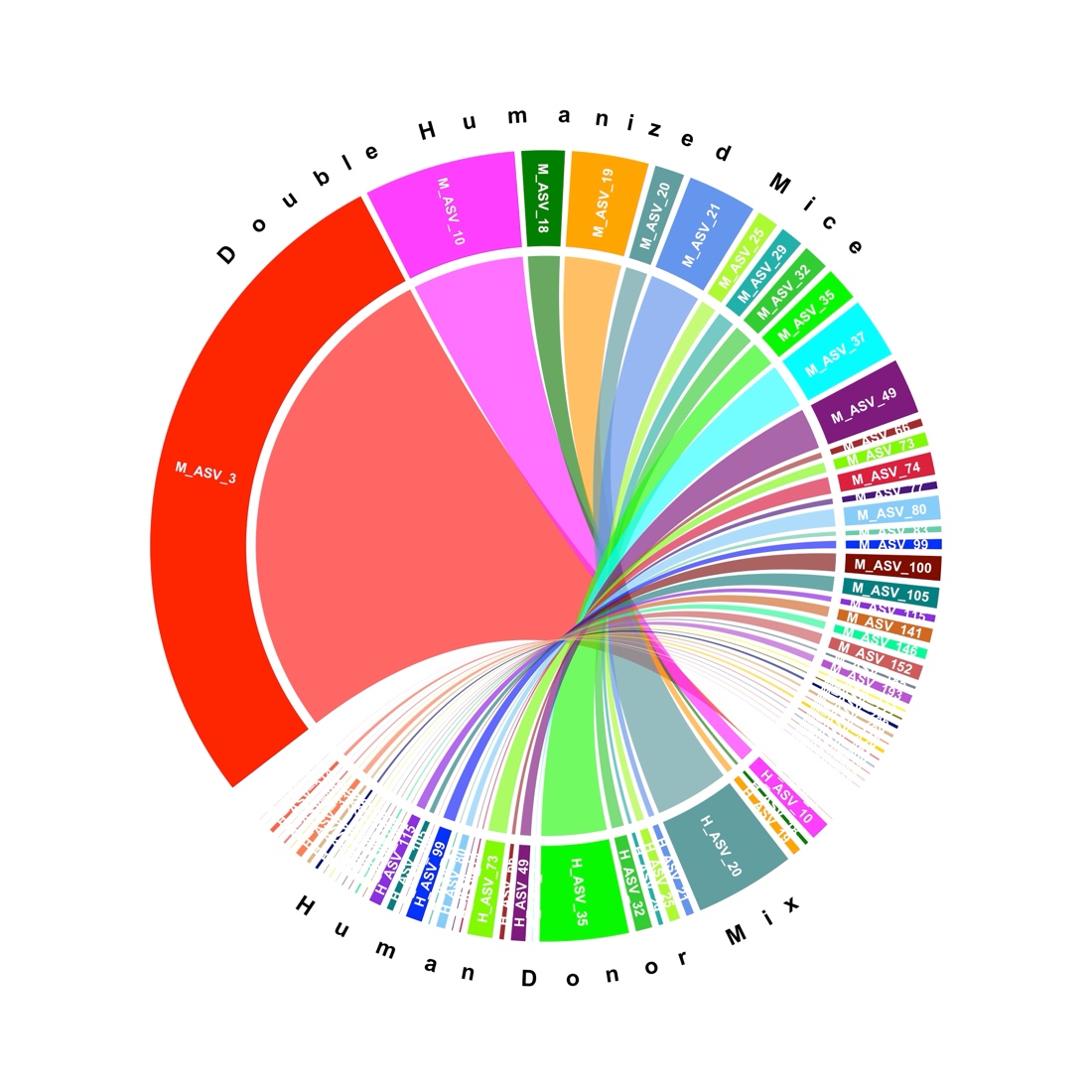


Figure S4: ASVs derived from the human donor mix that were consistently detected across all dHu-BLT mice two weeks post-FMT. Each sector represents an ASV (labeled 'H' for human donor mix and 'M' for mouse), with sector size reflecting the mean relative abundance of that ASV in the dHu-BLT mice and the human donor mix. The width of the connecting lines at each end indicates the respective abundance of the ASVs in the dHu-BLT mice and the donor mix.


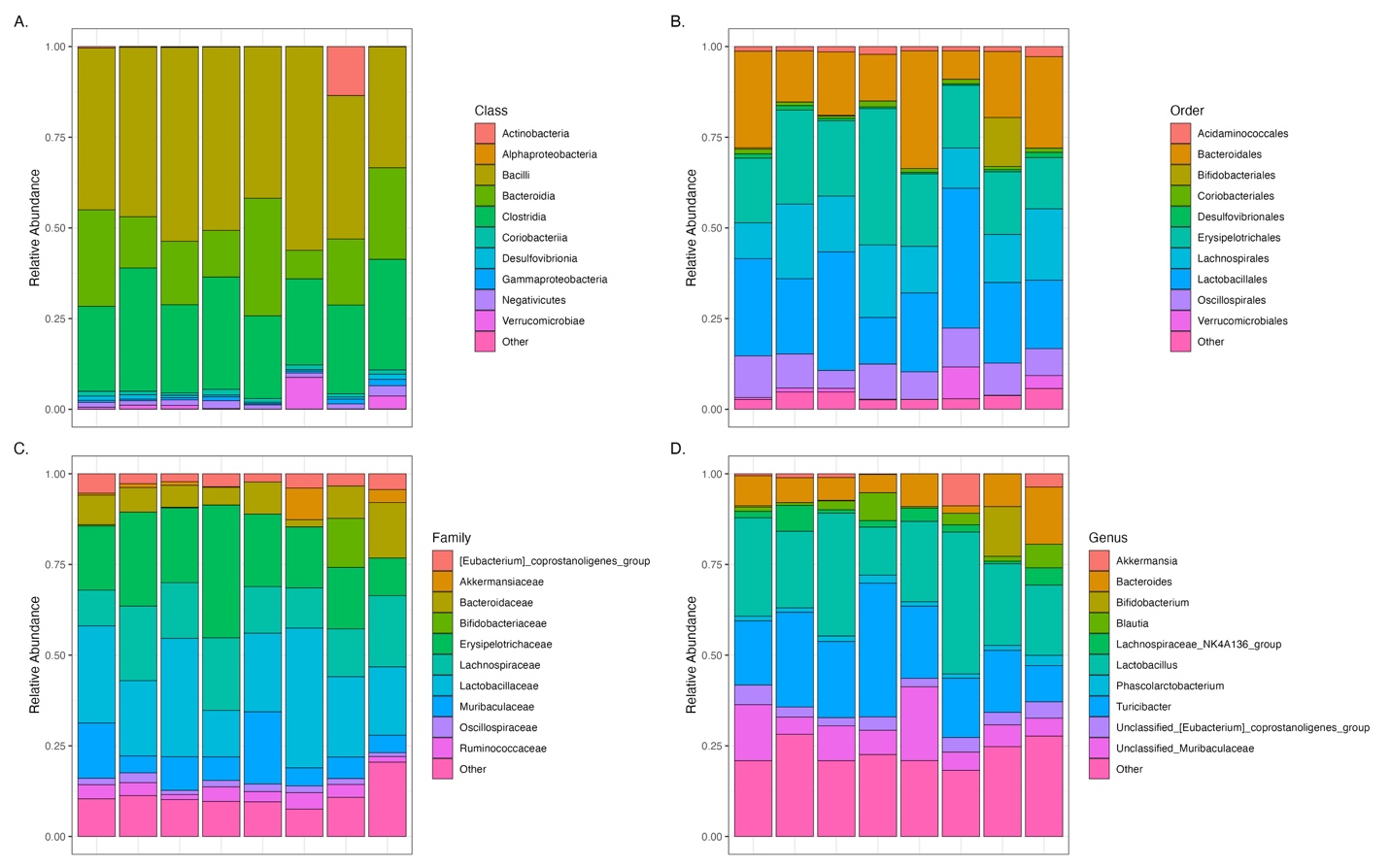


Figure S5: Microbial composition of dHu-BLT at different taxonomic levels. Relative abundance of different Class (A), Order (B), Family (C), and Genus(D) of dHu-BLT mice two weeks after a pool of healthy human microbiota (Donor mix) transplant. Each bar on the graph represents an individual mouse. Taxa outside the ten most abundant were grouped as “Other”.


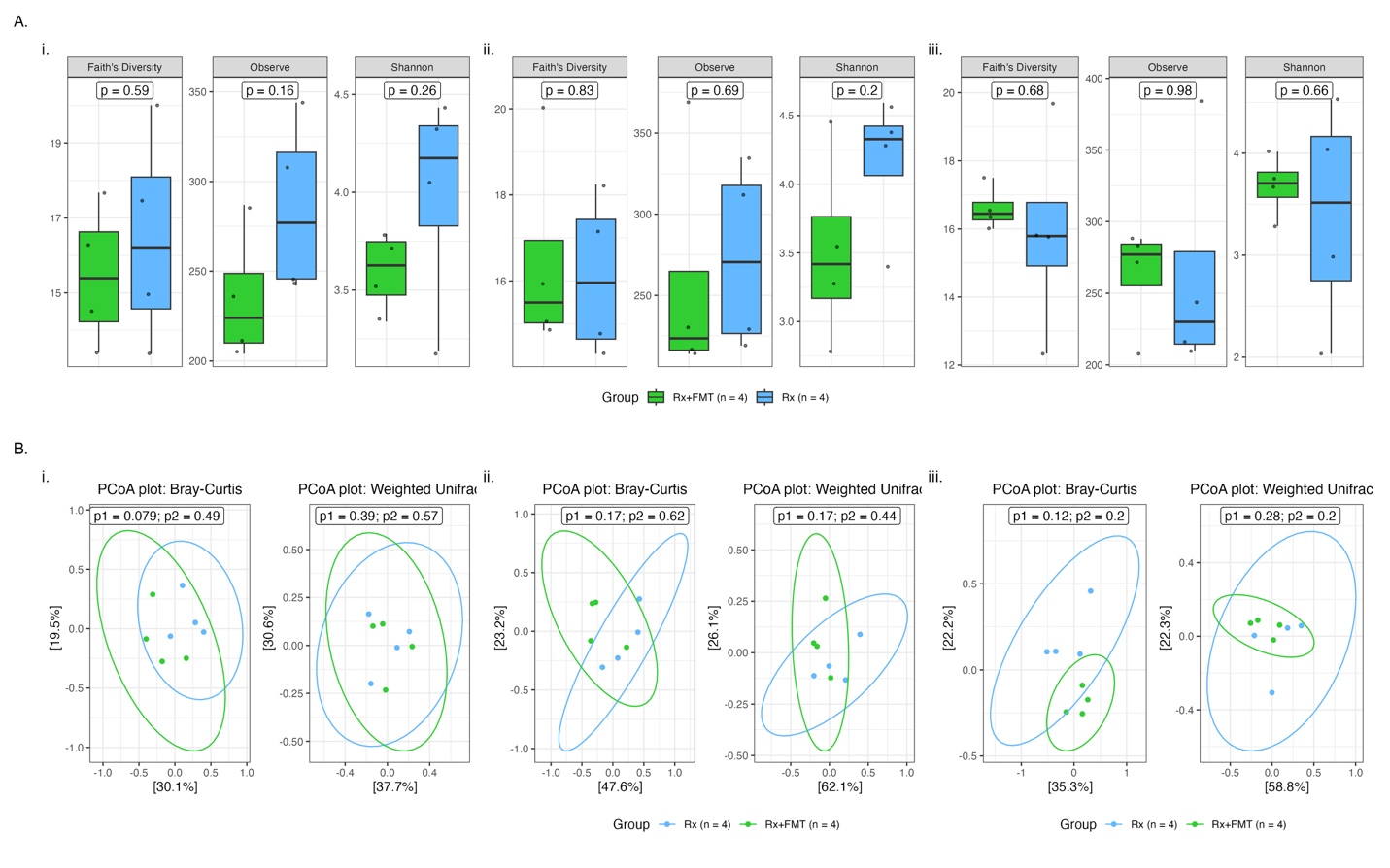


Figure S6: Alpha (A) and Beta (B) diversity at 10 days (i), 20 days (ii), and 30 days (iii) after FMT to one of the treatment groups. Each data point (A and B) represents an individual mouse. p = Welch’s t-test or Wilcoxon rank sums, p1 = Permutational Multivariate Analysis of Variance (PERMANOVA), and p2 = Permutational Analysis of Multivariate Dispersion (PERMDISP).
